# The dark side of SOX2: cancer - a comprehensive overview

**DOI:** 10.18632/oncotarget.16570

**Published:** 2017-03-25

**Authors:** Erin L. Wuebben, Angie Rizzino

**Affiliations:** ^1^ Eppley Institute for Research in Cancer and Allied Diseases, University of Nebraska Medical Center, Omaha, Nebraska, USA; ^2^ Department of Biochemistry and Molecular Biology, University of Nebraska Medical Center, Omaha, Nebraska, USA

**Keywords:** SOX2, cancer, tumor progression, tumor-initiating cells, cancer stem cell markers

## Abstract

The pluripotency-associated transcription factor SOX2 is essential during mammalian embryogenesis and later in life, but SOX2 expression can also be highly detrimental. Over the past 10 years, SOX2 has been shown to be expressed in at least 25 different cancers. This review provides a comprehensive overview of the roles of SOX2 in cancer and focuses on two broad topics. The first delves into the expression and function of SOX2 in cancer focusing on the connection between SOX2 levels and tumor grade as well as patient survival. As part of this discussion, we address the developing connection between SOX2 expression and tumor drug resistance. We also call attention to an under-appreciated property of SOX2, its levels in actively proliferating tumor cells appear to be optimized to maximize tumor growth - too little or too much SOX2 dramatically alters tumor growth. The second topic of this review focuses on the exquisite array of molecular mechanisms that control the expression and transcriptional activity of SOX2. In addition to its complex regulation at the transcriptional level, SOX2 expression and activity are controlled carefully by microRNAs, long non-coding RNAs, and post-translational modifications. In the Conclusion and Future Perspectives section, we point out that there are still important unanswered questions. Addressing these questions is expected to lead to new insights into the functions of SOX2 in cancer, which will help design novels strategies for more effectively treating some of the most deadly cancers.

## INTRODUCTION

The transcription factor Sox2 is widely recognized for its critical roles during mammalian embryogenesis. Although Sox2 was first shown to regulate the transcription of *FGF4* in mouse embryonal carcinoma cells [1], its importance was firmly established with the discovery that knocking out both alleles of *Sox2* results in embryonic lethality in mice. *Sox2* null embryos reach the blastocyst stage, but do not survive after implantation [2]. Shortly thereafter, knocking down Sox2 in mouse embryonic stem cells (ESC) was shown to disrupt their self-renewal and induce differentiation [3]. One year later, interest in Sox2 rose dramatically with the paradigm-shifting discovery by Takahashi and Yamanaka demonstrating conversion of mouse embryonic fibroblasts into induced pluripotent stem (iPS) cells by ectopic expression of Sox2 along with Oct4, Klf4, and cMyc [4].

The excitement surrounding the key roles of Sox2 in ESC and iPS cells, which are themselves tumorigenic, soon led to the search for SOX2 in cancer. Within a few years after the discovery of iPS cells, numerous reports of SOX2 expression in human cancer had already appeared. This soon turned into an avalanche of studies examining SOX2 in human cancer. The search terms “SOX2 and cancer” generate over 1,600 hits in the PubMed database and over 11,000 hits in PubMed Central. Since 2006, SOX2 has been implicated in growth, tumorigenicity, drug resistance, and metastasis in at least 25 different cancers, including cancers of the ovary, lung, skin, brain, breast, prostate, and pancreas (Tables 1-2). In the majority of these cancers, SOX2 has been reported to have increased expression or gene amplification in tumor tissue; however, the effects of SOX2 on tumorigenicity, prognosis, and drug resistance in human cancer have only begun to be explored. Nonetheless, it is evident from the impressive body of work published thus far that SOX2 is a major player in cancer and a potential therapeutic target.

**Table 1:** SOX2 expression and patient prognosis

Cancer Type	Amplified/ Increased Expression	Decreased Expression	Poor Prognosis/ High Tumor Grade	Good Prognosis/ Low Tumor Grade
Breast	Chen et al., 2008		Chen et al., 2008	
	Rodriguez-Pinilla et al., 2007		Piva et al., 2014	
Colorectal	Long et al., 2009		Saigusa et al., 2009	
			Lundberg et al., 2014	
			Talebi et al., 2015	
Embryonal (testicular germ cell) carcinoma	Biermann et al., 2007			
Esophageal	Gen et al., 2010		Wang et al., 2009	
	Bass et al., 2009			
	Long et al., 2009			
Ewing's sarcoma	Ren et al., 2016			
Gastric	Tian et al., 2014	Chen et al., 2016		Zhang et al., 2010
		Wang et al., 2015		Chen et al., 2016
		Otsubo et al., 2008		Wang et al., 2015
		Tsukamoto et al., 2005		
		Li X et al., 2004		
Glioblastoma	Alonso et al., 2011		Annovazzi et al., 2011	
	Schmitz et al., 2007		Ma et al., 2008	
	Phi et al., 2008			
	Annovazzi et al., 2011			
Head and neck squamous cell carcinoma	Bourguignon et al., 2012		Lee et al., 2014	Bayo et al., 2015
Hepatocellular carcinoma			Sun et al., 2013	
Lung adenocarcinoma	Sholl et al., 2010		Sholl et al., 2010	
Lung cancer, non-small cell			Chou et al., 2013	
Lung cancer, small cell	Güre et al., 2000			
	Rudin et al., 2012			
Lung cancer, squamous cell	Bass et al., 2009			Lu Y et al., 2010
	Hussenet et al., 2010			
	Yuan et al., 2010			
	Sholl et al., 2010			
	Wilbertz et al., 2011			Wilbertz et al., 2011
Lung cancer, neuroendocrine	Sholl et al., 2010			
Melanoma	Laga et al., 2011		Chen et al., 2013	
Nasopharyngeal carcinoma			Wang et al., 2012	
Oral squamous cell carcinoma	Freier et al., 2009		Du et al., 2011	
Ovarian	Belotte et al., 2015		Wang et al., 2014	Belotte et al., 2015
	Ye et al., 2011		Zhang et al., 2012	
	Zhang et al., 2012			
Pancreas	Sanada et al., 2006			
Prostate	Sattler et al., 2000		Kregel et al., 2013	
	Jia et al., 2011		Jia et al., 2011	
Sinonasal carcinoma	Schrock et al., 2013		Schrock et al., 2013	

**Table 2:** SOX2 expression in tumor-initiating cells, drug resistance, and tumor cell growth

Cancer Type	Cancer Stem Cells/Tumorigenicity	Drug Resistance	Alter Growth
Bladder	Hepburn et al., 2012	Hepburn et al., 2012	
Breast	Piva et al., 2014	Piva et al., 2014	Leis et al., 2012
	Simões et al., 2011		Chen et al., 2008
Cervical	Liu et al., 2014 *		
Colorectal	Lundberg et al., 2016		
Esophageal			Gen et al., 2013
			Bass et al., 2009
Ewing's sarcoma			Ren et al., 2016
Gastric	Tian et al., 2012	Tian et al., 2012	Hütz et al., 2013
		Tian et al., 2014	Tian et al., 2014
			Wang et al., 2015
Glioblastoma	Jeon et al., 2011	Hagerstrand et al., 2011	Fang et al., 2011
		Jeon et al., 2011	Cox et al., 2012
			Alonso et al., 2011
			Hagerstrand et al., 2011
			Gangemi et al., 2009
Head and neck squamous cell carcinoma	Lee et al., 2014 *	Lee et al., 2014	
	Bourguignon et al., 2012		
Lung adenocarcinoma	Nakatsugawa et al., 2011 *		
Lung cancer, non-small cell	Singh et al., 2012	Chou et al., 2013	Chou et al., 2013
	Xiang et al., 2011	Singh et al., 2012	
Lung cancer, small cell			Rudin et al., 2012
Lung cancer, squamous cell	Hussenet et al., 2010		Bass et al., 2009
			Hussenet et al., 2010
Medulloblastoma	Vanner et al., 2014 *		Cox et al., 2012
Melanoma	Santini et al., 2014 *		Laga et al., 2010
Osteosarcoma	Basu-Roy et al., 2015		Basu-Roy et al., 2012
Ovarian	Ma et al., 2010	Ma et al., 2010	Wang et al., 2014
	Yasuda et al., 2013	Yasuda et al., 2013	Yasuda et al., 2013
	Bareiss et al., 2013 *	Bareiss et al., 2013	
Pancreas	Herreros-Villanueva et al., 2013	Wuebben et al., 2016	Wuebben et al., 2016
Prostate	Rybak et al., 2013	Li et al., 2014	Cox et al., 2012
		Jia et al., 2011	Jia et al., 2011
Skin squamous-cell carcinoma	Boumahdi et al., 2014 *		

*Limiting cell dilution assays performed in connection with SOX2

In this review, we provide an overview of SOX2 in cancer and focus on two broad topics. The first part of the review discusses the expression and functions of SOX2 in cancer and specifically focuses on five main topics: 1) expression and amplification of SOX2 in cancer, 2) SOX2 expression and cancer prognosis, 3) SOX2 expression by cancer stem cells (tumor-initiating cells), 4) SOX2 and drug resistance, and 5) tight control of SOX2 expression in cancer. This last topic addresses what we believe is a defining feature of SOX2: its levels in actively proliferating tumor cells appear to be optimized to maximize tumor growth; namely, too little or too much SOX2 inhibits tumor cell proliferation. In the second part of the review, we delve into the exquisite levels of regulation used to ensure both proper expression and function of SOX2. Specifically, we focus on four main topics: 1) transcriptional regulation of SOX2, 2) regulation of SOX2 expression by microRNAs, 3) regulation of SOX2 expression by long non-coding RNAs, and 4) post-translational modifications of SOX2. In the final section of this review, Conclusions and Future Perspectives, we discuss several important questions that remain unanswered concerning the roles of SOX2 in cancer.

More generally, this review draws attention to four major themes. First, SOX2 plays important roles in many cancers. In at least three cancers, SOX2 has been shown to be expressed by the cancer stem cell/tumor-initiating cell population of the tumor. Second, there is growing evidence that SOX2 also influences the responses of tumor cells to drugs used in the treatment of cancer. Third, small changes in the levels of SOX2 alter tumor cell physiology. In several cancers, SOX2 expression increases during tumor progression, but, paradoxically, experimentally increasing SOX2 expression on its own in tumor cells with the aid of an inducible promoter leads to a reduction in growth. An explanation for this paradoxical finding is discussed. Fourth, a multitude of mechanisms have been shown to control the expression and function of SOX2. However, additional work will be needed to determine which of these mechanisms are utilized in specific cancers to fine tune SOX2 expression and function.

Finally, we recognize that other important topics are not included in this review. Specifically, we have not provided an overview of the downstream targets of SOX2 or the convergence of cell signaling and SOX2 expression. Similarly, we have not discussed the roles of SOX2 during embryogenesis and its roles in adult tissues. Readers interested in these subjects are directed to other recent reviews [5–7].

## EXPRESSION AND FUNCTION OF SOX2 IN CANCER

### SOX2 expression and amplification in cancer

SOX2 expression has been reported at both the RNA and protein levels for many cancers. Data available from The Cancer Genome Atlas indicates that *SOX2* mRNA is elevated in many cancers, relative to normal tissue. For example, *SOX2* is reported to be elevated in > 85% of glioblastoma multiforme samples compared to normal patient controls [8]. Interestingly, hypomethylation of the *SOX2* promoter was detected in over 250 glioblastoma specimens compared to normal patient controls [8]. In tumors such as glioblastoma, ovarian, esophageal, lung, oral, prostate, and sinonasal carcinoma, *SOX2* has been shown to be amplified in some subsets of patient tumors [8–19]. One study found *SOX2* to be amplified in 26% of serous ovarian cancers [9], and the *SOX2* locus (3q26.33) was amplified in ~8% of glioblastoma cases [8], indicating that an increase in copy number is part of the puzzle regarding *SOX2* expression in cancer.

For most cancers, SOX2 expression has also been documented at the protein level by immunohistochemistry [8, 10, 14–17, 20–32]. For example, in a study of breast cancer patients, SOX2 was strongly detected by immunohistochemistry in the nucleus of breast carcinoma cells compared to weak or no SOX2 staining in normal, non-tumorigenic mammary epithelial issue [20, 33]. Although SOX2 expression has been reported in many cancers [8–29, 32–36], the percent of SOX2-positive cells within SOX2-positive tumors has not been consistently reported. Additionally, in many studies, reference to “normal” tissue often was to unrelated, non-tumorigenic tissue rather than matched adjacent tissue.

In the case of ovarian cancer, both the percent of SOX2-positive tumors and the percent of SOX2-positive cells within these tumors have been reported [26]. Interestingly, the percent of SOX2-positive cells differs between different ovarian tumor subtypes [26]. In over 50% of cases of serous cystadenocarcinoma, SOX2 was expressed in > 75% of the cells of the tissue examined, whereas in only 5% of cases of less severe serous cystadenoma was SOX2 expressed in > 75% of the cells. Similar expression patterns were observed with mucinous epithelial lesion [26]. Interestingly, this variable expression of SOX2 across cells within the same tumor has been observed in multiple cancers [37, 38] and may influence their physiology, as discussed below in the section “SOX2 and Tumor-Initiating Cells/Cancer Stem Cells”. Finally, it should be stressed that comparisons of SOX2 expression between tumor cells and normal tissues may be misleading, because tumor cells arise from a small subset of cells in a tissue and the expression of SOX2 in this subset of cells may differ from that in the remainder of the tissue.

For several cancers, the levels of SOX2 expression at different stages of the cancer have been examined [25]. In pancreatic ductal adenocarcinomas (PDAC), SOX2 is rarely expressed cases of in pre-malignant pancreatic intraepithelial neoplasia, but its expression has been reported to increase to ~60% in cases of poorly differentiated and neurally invasive components [28]. Similarly, studies of glioblastoma, esophageal, breast, and prostate cancers have reported that SOX2 levels increase with increasing tumor grade [14, 29, 33, 39, 40], and for prostate cancer the percentage of SOX2-positive cells correlates with Gleason score [41]. In the case of ovarian epithelial carcinoma, SOX2 expression was reported to increase from ~55% of normal ovarian epithelia samples expressing some SOX2 (in a relatively low percent of cells) to over 90% of serous and mucinous cystadenocarcinomas samples expressing SOX2 and in a much higher percent of the cells [26]. Interestingly, in the case of gastric cancer, reports regarding the levels of SOX2 expression during tumor progression are conflicting. In one study, *SOX2* mRNA was reported to be significantly elevated compared to adjacent benign tissues [34]. In contrast, other studies reported lower SOX2 expression in gastric cancer and its metastatic lesions compared to matched, normal gastric mucosa [30, 31, 42, 43]. Notably, SOX2 expression also appears to vary with different mucosal subgroups in gastric cancer [30, 44]. Thus, for several cancers, there is a need to more carefully determine how SOX2 levels change during tumor progression. Recognizing how SOX2 expression is altered between normal and tumorous tissues is important for understanding molecular changes necessary for tumor initiation and progression.

### SOX2 expression: prognosis and survival

In addition to determining how SOX2 levels change during tumor progression, it is essential to determine whether SOX2 levels correlate with clinical prognosis for cancer patients. Studies reported thus far indicate that high SOX2 levels correlate with poor prognosis for patients with many different cancers, including breast, colorectal, esophageal, ovarian, prostate, and some lung tumors, as well as nasopharyngeal and sinonasal carcinoma (Table 1) [27, 38, 40, 45–48]. Furthermore, a higher incidence of recurrence was correlated with *SOX2* amplification in sinonasal carcinomas [19], and rectal cancer patients with elevated SOX2 displayed significantly shorter disease-free survival following chemoradiotherapy [45]. Studies in esophageal, hepatocellular, oral/tongue and some lung cancers have also found a correlation between elevated SOX2 and decreased survival [27, 40, 48–52]. In addition to survival and recurrence, in the majority of cancers examined thus far, high SOX2 expression has been linked to the infiltrative and metastatic capacity of tumor cells [31, 50, 53–56]. For example, in the cases of colorectal cancer and prostate cancer, SOX2-expressing tumors have been shown to correlate with increased distant and lymphatic metastases [29, 53]. Similarly, in esophageal squamous cell carcinomas, tumors in which more than 50% of the cells express SOX2 were correlated with increased lymphatic and vascular invasion, poor differentiation, and incomplete surgical resection [55].

Consistent with many reports linking SOX2 expression to increases in metastasis, a strong connection between SOX2 and epithelial-mesenchymal transition (EMT) has been established in many tumor types, including colorectal, esophageal (ESCC), laryngeal, pancreatic, lung (NSCLC), gastric, breast, and prostate cancer [54, 57–63]. Additionally, high SOX2 expression has been linked to increases in migration and invasion [54, 57, 58]. The link between SOX2 and EMT in some cancers was shown by directly altering the levels of SOX2. For example, knockdown of SOX2 in colorectal tumor cells induced mesenchymal-epithelial transition [54]. In other studies, changes in SOX2 expression and EMT were observed in response to changing other factors. For example, in pancreatic cancer, expression of the transcription factor NFATc1 was reported to drive EMT *via* SOX2-dependent transcription [59].

Although SOX2 is associated with poor prognosis in many cancers, high SOX2 levels may not be uniformly indicative of poor patient prognosis. For at least four cancers, including gastric cancer and squamous cell lung cancer, low SOX2 expression has been reported to correlate with poor prognosis (Table 1) [16, 31, 56, 64]. Moreover, as noted earlier, some studies of gastric cancer indicate that elevated SOX2 levels are linked to reduced lymph node and distant metastases. The reasons for the contrasting results for SOX2 levels in different cancers remain to be determined.

Disappointingly, for some cancers, in particular head and neck squamous cell carcinomas and ovarian cancer, there are conflicting reports regarding the levels of SOX2 expression and patient survival [9, 49, 52, 65]. In the case of head and neck squamous cell carcinomas, initial studies by Lee et al showed that SOX2 expression is correlated with poor prognosis and a nearly 5-fold higher risk of recurrence [49], but subsequent studies by Bayo et al determined that SOX2^high^ tumors had a median progression-free survival of 51 months compared to SOX2^low^ tumors (16 months) and that SOX2^high^ tumors had a > 110 month improved overall survival compared to SOX2^low^ tumors [65]. Questions also exist in the case of ovarian cancer. Belotte et al reported that tumors with *SOX2* amplification had statistically significant improved survival [9]; however, an earlier study from Wang et al reported that high SOX2 levels in both primary and metastatic tumor components statistically correlated with significantly worse survival [52]. It is evident from the discussion in this section that there is a clear need for further investigation into the clinical implications of SOX2 expression, particularly how SOX2 levels influence tumor progression and patient survival.

### SOX2 and tumor-initiating cells/cancer stem cells

SOX2 is not only expressed in many types of cancer, it has also been implicated in the tumor-initiating populations (proposed cancer stem cell population) of many of these tumors (Table 2). Many studies have used putative cancer stem cell markers, such as CD133, CD44, ABCG2, and side population *via* Hoechst efflux assay, to isolate and enrich for cells capable of forming tumors [tumor-initiating cells (TIC)] [13, 35, 66–72]. For example, in the case of an ovarian cancer cell line, the side population exhibited elevated levels of *SOX2* mRNA and a higher percentage of TIC when assayed using a limiting cell dilution tumor assay, the gold standard for assessing the frequency of TIC within a tumor [69]. However, for most cancers, the link between SOX2 and their TIC has not been firmly established. For several tumor types, knockdown of SOX2 and/or ectopic expression of SOX2 have been used to implicate SOX2 in the biology of the TIC [38, 73–77]. For example, Lee et al and Santini et al determined that stable knockdown of SOX2 in limiting cell dilution tumor assays dramatically reduced tumor initiation/formation in both head and neck squamous cell carcinomas and melanomas, respectively [49, 78]. Conversely, others generated lung and ovarian tumor cells that stably overexpress SOX2 and reported an elevated number of TIC when these cells were tested in limiting cell dilution tumor assays [79, 80]. However, as discussed later in the review, there are concerns about the use of tumor cells engineered to stably overexpress SOX2. Additionally, we raise concerns about the use of putative stem cell markers for the isolation of TIC/cancer stem cells.

Arguably the most conclusive studies have linked SOX2 to TIC by isolating the SOX2-positive cell subpopulation rather than experimentally altering the levels of SOX2 within cells. This is important, because SOX2 is expressed heterogeneously throughout the cells of many tumors [37, 38, 65, 81]. Moreover, for some tumors, only a small percentage of the cells express SOX2. This is particularly evident for SOX2-positive tumor cell lines [37, 38, 82]. Thus far, the SOX2-positive cells isolated from heterogeneous populations were engineered to express either GFP that was knocked into the endogenous *SOX2* gene (*GFP* coding sequences replaced single *SOX2* exon) [37, 82] or GFP driven by a transgene under the control of the *SOX2* promoter and enhancer [83]. In these three studies, SOX2-positive cells exhibited a higher frequency of TIC compared to the SOX2-negative cells of the same tumor cell population in a limiting cell dilution tumor assay. Furthermore, studies by Vanner et al showed that the rare SOX2-positive cells are members of a quiescent, slowly-cycling cancer stem cell population that repopulates the tumor when cytotoxic drugs are withdrawn [37]. Similarly, a recent study in bladder cancer has also shown that the quiescent label-retaining cancer stem cell population does not respond to cytotoxic therapy and is capable of repopulating the tumor following drug removal [84]. Such studies not only call attention to a role for SOX2 in the TIC population, but also that this slowly-cycling, SOX2-positive population may be responsible for repopulating the tumor after drug treatment is suspended.

### Cancer stem cell markers - a cautionary note

As discussed earlier, there is compelling evidence that SOX2 is associated with the tumor-initiating population of at least three cancers and there is growing evidence for this association in many other cancers (Table 2). However, as we explain below, caution should be exercised when putative stem cell markers (e.g. CD133 and ALDH1) that change rapidly in response to cellular conditions are used to isolate cancer stem cells. To illustrate this point, we focus on CD133, which has been linked with SOX2 expression and cancer stem cells in many tumor cell types [85–91]. In 2003, CD133 was reported to serve as a marker for the tumor-initiating cells of brain tumors. Specifically, it was reported that the capacity for self-renewal of brain tumor cells in culture (non-adherent tumor spheres) resides in the CD133^+^ cell population and not in the CD133^-^ population [92]. Subsequently, it was reported that transplantation of as few as 100 CD133^+^ glioblastoma cells or 1,000 CD133^+^ medulloblastoma cells were sufficient for tumor formation; whereas, 10^5^ CD133^-^ glioblastoma cells or 5×10^4^ CD133^-^ medulloblastoma cells were incapable of forming tumors [93]. The work with CD133 in brain tumors was followed by a series of studies supporting the value of CD133 as a marker for identifying and isolating tumor-initiating cells in several other cancers, including pancreas, prostate, lung, liver, and colon tumors [94–99]. However, the use of CD133 as a stem cell marker for several cancers, including pancreatic ductal adenocarcinoma and glioblastoma, has become controversial [22, 100]. In the case of glioblastoma, one study reported that both CD133^-^ and CD133^+^ cells were able to induce tumors in nude mice, although tumors formed by the CD133^-^ cells exhibited a lower level of proliferation [100].

We believe the conflicting results reported for CD133^-^ cells, which has been noted by others [101], is due at least in part, if not entirely, to rapid changes in expression of CD133 in response to cell culture conditions. For example, the percentage of CD133^+^ DAOY medulloblastoma cells increases when they are switched from 20% to 2% oxygen [102] and there is a large increase in the expression of CD133 when CD133^-^ cells are switched from serum-containing medium to serum-free medium [103]. Similarly, elevating SOX2 in medulloblastoma cells, which causes a major reduction in their growth, leads to over a 10-fold increase in the number CD133+ cells just one day after elevating SOX2 [104]. Additionally, we have observed that cell surface expression of CD133 on DAOY medulloblastoma cells increases (~50 to 90%) as cell density increases (Wilder and Rizzino, unpublished results). Importantly, we have also shown that CD133 expression rapidly reappears when freshly isolated CD133^-^ glioblastoma cells are returned to cell culture [104]. Similarly, we have observed the reappearance of ALDH1^+^ cells when freshly isolated ALDH1^-^ pancreatic ductal adenocarcinoma cells were returned to cell culture (Wilder and Rizzino, unpublished results). Clearly, if stem cell markers fluctuate in respond to cell culture conditions, it is not surprising that conflicting results regarding CD133 and ALDH1 have been reported.

If CD133 is not a bonafide marker of tumor-initiating cells, why has it been reported in many studies to enrich for putative cancer stem cells? Although the answer to this question remains to be determined fully, three findings suggest an explanation. First, CD133 has been shown to influence MAPK/ERK signaling in brain tumor cells [105]. Second, the 52-amino acid C-terminal cytoplasmic tail of CD133 can be phosphorylated by a Src-family member on two tyrosine residues (Y828 and Y852) in medulloblastoma cell lines [106]. Third, N-linked glycosylation of CD133 is decreased when colon tumor cells are treated with butyrate [107]. Thus, CD133 appears to be involved in signal transduction, and it may help cells sense and respond to changes in the extracellular environment. Interestingly, this is highly reminiscent of the discovery that the transmembrane glycoprotein MUC1, which was initially identified as a tumor-associated antigen, plays an important role in signal transduction. In this regard, the cytoplasmic C-terminal tail of MUC1 can be phosphorylated by different kinases, including EGFR, PDGFR-β, and MET. Remarkably, phosphorylation of the C-terminal tail of MUC1 by MET and PDGFR-β induces its translocation into the nucleus and increases the metastasis of pancreatic ductal adenocarcinoma cells, respectively [108–110].

Together, the studies discussed above, which point to the role of CD133 in signal transduction, along with the studies demonstrating rapid modulation of CD133 when cell culture conditions are altered, lead us to propose that CD133 is part of the essential stress response machinery that enables tumor cells to rapidly adapt to a range of highly detrimental conditions, such as hypoxia, when transplanted either subcutaneously or orthotopically. If, in fact, expression of CD133 and other stem cell markers provides protection against a range of harmful conditions, this could explain why cells that express these markers exhibit a higher frequency of tumor-initiating cells: they are primed to handle the stress. Importantly, if the expression of putative stem cell markers, such as CD133, provides protection against the stresses of transplantation, their use for isolation of cancer stem cells may lead to an overestimation of the number of TIC, especially when performing limiting cell dilution tumor assays. Thus, we suggest, wherever possible, that future efforts to associate SOX2 with cancer stem cells of a given type of tumor focus on those markers that do not change readily in response to changes in cellular condition. As discussed above (SOX2 and Tumor-Initiating Cells/Cancer Stem Cells), this problem was avoided in those studies where GFP had been knocked into the endogenous SOX2 locus, and GFP^+^ cells were isolated and tested for enrichment of tumor-initiating/cancer stem cells.

### SOX2 and drug resistance

Several recent studies have shown that exogenous elevation of SOX2 can promote resistance to chemotherapeutics currently being used clinically [29, 34, 38, 48, 49, 67, 75, 80, 111–114]. In a report from Bareiss et al, ovarian cancer cell lines that did not express SOX2 and that were sensitive to carboplatin, cisplatin, and paclitaxel became resistant following stable, ectopic expression of SOX2 [80]. Furthermore, in a SOX2-expressing ovarian cancer cell line, SOX2 knockdown using short hairpin RNAs (shRNA) provided susceptibility to these drugs, which was reversed upon re-expression of SOX2 ectopically [80]. Similar results were seen in breast cancer cell lines, as stable overexpression of SOX2 in MCF-7 cells promoted resistance to tamoxifen, while stable downregulation of SOX2 using shRNAs enhanced the sensitivity of MCF-7 cells to tamoxifen [38]. Additionally, stable overexpression of SOX2 in PC3 prostate cancer cells promoted evasion of apoptosis in cells treated with paclitaxel [113]. Furthermore, it has been shown that inducible-overexpression and inducible-knockdown of SOX2 in PDAC cell lines altered responses to small molecule inhibitors targeting MEK and AKT signaling. In this study, overexpression of SOX2 protected PDAC cells from the growth inhibitory effects of MEK and AKT inhibitors; whereas, knocking down SOX2 enhanced the growth inhibition in the presence of these drugs [114]. While SOX2 may be acting to protect tumor cells through antiapoptotic signaling or quiescent-like phenotypes [29, 37, 67, 75], SOX2 may also be promoting drug resistance through various ATP-binding cassette (ABC) transporters, including ABCG2, ABCC3, and ABCC6. In particular, ABCG2 has been shown in various tumors to be upregulated in the side population TIC [66, 69] and, in some tumors, is considered to be an additional cancer stem cell marker. Furthermore, studies in lung cancer, as well as head and neck squamous cell carcinoma, have shown that stable downregulation of SOX2 *via* shRNAs decreases ABCG2, which implicates this transporter in SOX2-related drug resistance [49, 76]. Recognizing and focusing on the role of SOX2 in drug resistance could greatly improve the treatment options for patients with a multitude of cancers, especially those with highly refractory tumors, as the ability to eradicate the TIC population is likely to be the only way to prevent recurrence.

### SOX2 levels and tumor growth

Many studies have used stable overexpression and/or knockdown of SOX2 in tumor cell lines to better understand the roles of this transcription factor in cancer. Knockdown of SOX2 using either small interfering RNA (siRNA) or shRNA have been used in multiple studies [8, 11, 12, 25, 29, 32–34, 48, 52, 69, 111, 114–118]. Importantly, even partial reductions in SOX2 levels have been reported to significantly decrease cell viability, clonal growth, sphere formation, and tumorigenicity in multiple cancer types. Clearly, knockdown studies have established that SOX2 plays important roles in these cancers. However, SOX2 overexpression studies have generated conflicting results. For example, stable overexpression of SOX2 in the gastric tumor cell line N87 was reported to increase growth both *in vitro* and *in vivo* [34]. In contrast, stable overexpression of SOX2 in the gastric cell line MKN28 was reported to decrease growth both *in vitro* and *in vivo*. Currently, the reasons for the conflicting results are unclear. In both studies, SOX2 was substantially overexpressed in gastric tumor cell lines that endogenously express relatively little SOX2. Part of the explanation may be due to differences in the cell lines used. However, as discussed below, other factors related to experimental design may also be a contributing factor.

Conflicting reports from SOX2 overexpression studies have also been reported for breast, prostate, and pancreatic cancers. Stable overexpression in MCF-7 (breast), DU145 (prostate), and Patu8988t (PDAC) cells have been reported to increase growth *in vitro* [29, 33, 70]. However, there are reports showing that elevating SOX2 does not promote growth. In the case of three colorectal cancer cell lines, growth inhibition was observed during the initial five days when SOX2 was elevated. In the case of HT-29, growth is almost completely arrested when SOX2 was elevated [119]. Moreover, overexpression of SOX2 from a doxycycline inducible transgene demonstrated that overexpression of SOX2, where one can monitor the early, short-term consequences of elevating SOX2, does not increase cell proliferation. Inducibly elevating SOX2 (~5 to 7-fold) in glioblastoma (U87, U118), medulloblastoma (DAOY), breast carcinoma (MDA-MB-231), and prostate carcinoma (DU145) cell lines led to growth inhibition in each case. More recently, the same results were observed in three different PDAC cell lines [114]. Elevating SOX2 (~5 to 7-fold) in each of these cell lines with the aid of an inducible promoter led to growth inhibition *in vitro*. Furthermore, the effects of elevating SOX2 *in vivo* have also been examined. Initially, we determined that elevating SOX2 with the aid of an inducible promoter in one of these PDAC cell lines dramatically reduced tumor growth [114]. More recently, we have observed the same effect on tumor growth when SOX2 was elevated in a second PDAC cell line (Wuebben and Rizzino, unpublished results). Thus, the effect of elevating SOX2 in these tumor cell lines, in particular PDAC tumor growth, is growth inhibition. Going forward, it will be important to reexamine the effects of SOX2 in other cancers using inducible overexpression of SOX2, in particular where stable overexpression has been reported to increase tumor cell growth.

The contrasting results obtained from studying cells in which SOX2 was stably overexpressed *versus* inducible overexpression of SOX2 are likely to result from the fundamental difference in experimental design. Cell lines engineered for inducible overexpression of SOX2 were generated *via* drug selection of lentiviral transduced cells, which occurred at frequencies greater than 70%, before SOX2 levels were altered. In direct contrast, establishing cells lines that stably overexpress SOX2, which takes several weeks, involves drug selection at a time when SOX2 levels are also ectopically elevated. As a result, any cells that are unable to grow or grow more slowly due to elevated levels of SOX2 will be lost during the drug selection period as the faster proliferating cells expand. Consequently, the cells present in the drug selected population represent only a subpopulation of the parental cells.

It is evident from the studies where SOX2 was elevated from an inducible transgene that many, if not most, SOX2-expressing tumor cell lines are growth inhibited when SOX2 is suddenly elevated. However, this does not mean that SOX2 levels cannot rise during cancer. In fact, several lines of evidence argue that SOX2 levels do rise during cancer. As discussed earlier, the *SOX2* gene is amplified in several cancers, and SOX2 has been shown to be expressed in some tumors, but not in their surrounding tissue. Moreover, in some tumors, SOX2 expression has been shown to increase during tumor progression [14, 26, 29, 33, 39–41]. We suggest that two factors, which are not mutually exclusive, are likely to contribute to the apparent increase in SOX2 expression during tumor progression. First, there may be an increase in the number of SOX2-positive cells in the tumor population. If SOX2 is required for the tumor-initiating/cancer stem cell population, which is the case for at least some cancers, SOX2 levels may rise as the population of TIC increases during tumor progression. Second, increases in SOX2 levels in individual cells may contribute to tumorigenicity, but only when accompanied by corresponding changes in the expression of other genes that counterbalance the growth inhibitory effects of elevated SOX2. In this regard, two recent studies have shown that mutations in RB1 and p53, which occur in high risk prostate cancer patients, leads to significant elevation of SOX2 in an animal model of prostate cancer and in the androgen dependent prostatic tumor cell line LNCaP [120, 121]. Thus, the effects of SOX2 appear to be highly context-dependent, similar to other genes, notably TGFβ, which can act as a tumor suppressor or oncogene. Furthermore, we propose that identifying genes that permit SOX2 to contribute to tumorigenicity provides a novel strategy for identifying new therapeutic targets that can block the growth of SOX2-dependent tumors. In this regard, blocking the expression or function of genes that enable elevated SOX2 levels to promote tumor growth could convert the action of SOX2 from a growth promoter (oncogene) to a growth inhibitor (tumor suppressor gene). Given the difficulty of targeting transcription factors directly, this should be given serious consideration.

### Exquisite control of SOX2 levels

The discussion in the previous section deals with an important property of SOX2 in cancer: its levels must be carefully controlled. This delicate balance was first identified in ESC. Both the knockdown of SOX2 and its overexpression block the self-renewal of ESC and lead to their differentiation [3, 122]. Remarkably, a 2-fold increase in SOX2, from an inducible transgene, is sufficient to rapidly induce the loss of self-renewal and trigger the differentiation of ESC [122]. More recently, we have shown that inducible overexpression of SOX2 in multiple tumor cells, including three PDAC cell lines, leads to growth inhibition [104, 114]. In the case of PDAC cells, knockdown as well as the overexpression of SOX2 in PDAC cell lines strongly reduces their growth both *in vitro* and *in vivo* [114]. Accordingly, we propose that SOX2 levels in actively proliferating tumor cells are optimized to maximize tumor growth: too little or too much SOX2 decreases tumor growth and alters cell behavior [104, 114].

The need to carefully control the levels of SOX2 raises a fundamental question, why do small changes in the levels of SOX2 exert such a profound change in cellular function? This question has been addressed in earlier review articles, which discussed why small changes in SOX2 levels disrupt the self-renewal and pluripotency of ESC [6, 123]. Therefore, only the main points are summarized here. Unbiased proteomic screens of the Sox2-interactome and genome wide binding studies of Sox2 in mouse ESC demonstrate that Sox2 is part of a highly interdependent transcriptional network that is interconnected at multiple levels [124]. Moreover, proteomic analysis of ESC undergoing differentiation indicate that the SOX2-interactome changes when ESC initiate differentiation [124, 125]. Analysis of the Sox2-interactome in ESC demonstrates that Sox2 physically associates with many other master regulators in ESC, including Oct4, and these master regulators associate with many of the same nuclear proteins. This creates a highly integrated transcriptional network. As predicted, knockdown of proteins that associate with Sox2 and other master regulators in ESC leads to the loss of self-renewal and pluripotency [125–127]. More surprising was the finding that Sox2 associates with > 50% of the genes that code for Sox2-associated proteins [124]. Furthermore, SOX2 associates with proteins in ESC that have prominent roles in signal transduction and DNA repair and replication. This is not only true in ESC, SOX2 has also been shown to associate with a diverse array of functionally distinct proteins in brain tumor cells [128, 129]. Thus, it is not surprising that small changes in SOX2 levels exert such profound effects over cell physiology. Given the need for careful regulation of SOX2, its expression must be controlled at many levels. In the next section, we describe an exquisite array of mechanisms that are used to precisely control the expression and function of SOX2.

## REGULATION OF SOX2 EXPRESSION

### Transcriptional regulation of SOX2

The *SOX2* gene in mammals, as well as birds, is located within a gene desert (a large genomic region largely devoid of other protein coding genes). Analysis of a 200 kb region of the chicken gene that surrounds the *SOX2* single exon identified at least 27 distinct enhancers that are transcriptionally active for the regulation of *SOX2* during neuro-sensory development in the chicken [130]. Eleven of the enhancers are distributed fairly evenly over a 97 kb region located upstream of the coding region of the *SOX2* gene, and 16 enhancers are fairly evenly distributed over a 110 kb region downstream of the coding region of the *SOX2* gene The large majority of the enhancers identified in the chicken genome are located in regions that are conserved in mammals [130]. Thus, it is likely that the mammalian *SOX2* gene is also transcriptionally regulated by a large number of distinct distal enhancers during different stages of development. However, far more work will be needed to define the regulatory regions of mammalian *SOX2* gene that are active in specific cell types. As discussed below, only three enhancers have been identified as functionally active in mammalian cells, one of which is located ~100 kb downstream of the *Sox2* gene.

In mammalian cells, transcriptional regulation of the *Sox2* gene, including enhancers that drive *Sox2* expression, has been primarily studied in mouse ESC. In addition to the basal promoter of the *Sox2* gene [131], early studies identified two enhancers, *SRR1* and *SRR2*, which influence the activity of the *Sox2* promoter [132]. *SRR1* is located ~4kb upstream of the *Sox2* transcription start site; whereas, *SRR2* is located ~2.5 kb downstream of the 3’ end of the *Sox2* coding region. Although *SRR1* has been shown to be active in promoter/reporter gene constructs expressed in ESC, its impact on the expression of *Sox2* in ESC is minimal when *SRR1* is deleted from the endogenous *Sox2* gene [133]. However, deleting a region -5.7 to -3.3 kb upstream of the *Sox2* transcription start site, which contains *SRR1*, abolished expression of *SOX2* in telencephalic neural stem cells and precursors during murine development [134, 135]. *SRR2* is not only active in mouse ESC; it has been used to isolate human iPS cells [136]. For these studies, the *SRR2* enhancer was multimerized (4 tandem repeats) and inserted into a lentiviral vectors which drives the expression of enhanced green fluorescence protein (EGFP) *via* a minimal promoter only when *SRR2* is active. Subsequently, this lentiviral vector which drives EGFP was shown to be active in breast cancer cells [115]; and isolation of the subset of EGFP-expressing breast tumor cells were shown to exhibit enhanced tumorigenic potential, but, unexpectedly, only when NOD/SCID mice were engrafted with a large number of cells [137].

Several studies have examined the transcriptional machinery that regulates the activity of *SRR2*. The sequence of *SRR2* contains adjacent *HMG* and *POU* motifs (referred to as an *HMG/POU* cassette) that have been shown to be essential for the activity of *SRR2* in ESC and bind Sox2 and Oct4 in ESC [132, 138]. These studies led to the conclusion that Sox2 in combination with Oct4 contributes to the transcription of *Sox2* in ESC. However, this may not be the only role of *SRR2* in the transcription of *Sox2*. In fact, several recent studies lead us to suggest that *SRR2* may also repress *Sox2* transcription, especially during differentiation. First, as in the case of *SRR1*, deletion of *SRR2* from the endogenous *Sox2* gene did not significantly reduce *Sox2* expression in ESC [133]. Even more suggestive of a repressive role for *SRR2* is the finding that *SRR2* is able to bind transcriptional repressors, such as p21, p27^Kip1^, and the p130/E2F4-SIN3A repressor complex, in neural stem cells and iPS cells undergoing differentiation [139, 140]. Consistent with these findings, *Sox2* mRNA is elevated in *Rb* (p105) null and *p130* (retinoblastoma family member) null MEFs and it is elevated in the pituitary tissue of *Rb* heterozygous mice [141]. Moreover, in pituitary tumors, loss of *Rb* or *p130* has been linked to a defect in the repression of *Sox2* expression [141]. Given the roles of p21, p27^Kip1^, and Rb proteins in the G1 cell cycle check point, *Sox2* expression may be reduced in the G1 phase of the cell cycle. Thus, future studies should consider whether *SOX2* expression is cell cycle regulated.

In ESC, a critical enhancer region (referred to as *SCR* - *Sox2* control region) that is required for *Sox2* transcription is located ~100 kb downstream of the *Sox2* gene [133]. Previous studies predicted 10 enhancers surrounding the *Sox2* gene, including two that overlapped *SRR1* and *SRR2*. When tested in promoter/reporter gene constructs, three of the 10 putative enhancers, which are located 18, 107, and 111 kb downstream of the *Sox2* gene, were found to drive the expression of the reporter gene more potently than *SRR1* and *SRR2* in ESC. More definitive results were obtained by generating deletions of these enhancers in one allele of the SOX2 gene using CRISPR based gene editing [133]. Deletion of *SRR1*, *SRR2*, or the enhancer located 18 kb downstream of SOX2 did not affect the expression of the targeted allele. In strong contrast, deletion of the SCR reduced expression of the targeted allele. (For these studies, expression of the targeted and non-targeted alleles was monitored separately by PCR in a heterozygous ESC line containing one allele from mouse strain *Mus musculus* and one allele from *Mus castaneus*.) Notably, targeting one *Sox2* allele in ESC did not impact the maintenance of pluripotent ESC, due to upregulation of the non-targeted *Sox2* allele. This finding and earlier studies involving Sox2 overexpression in ESC (see below) indicate that Sox2 influences its own expression in ESC by a feedback loop. Going forward, it will be important to determine whether the *SCR*, which is active in ESC, is also active in other SOX2-expressing cells, in particular SOX2-positive tumor cells. Thus far, only *SRR2* has been reported to be active in SOX2-positive tumor cells.

Sox2 not only positively influences Sox2 expression in ESC when it is under expressed, it has the opposite effect when Sox2 is overexpressed in ESC. As mentioned earlier in this review, ESC engineered for inducible overexpression of Sox2 undergo differentiation when Sox2 is elevated by 2-fold or more. Interestingly, overexpression of exogenous Flag-tagged Sox2 in ESC reduces expression of endogenous Sox2 at the protein level [122] and at the transcriptional level [142]. Specifically, it was determined that elevation of exogenous Sox2 activated a negative feedback loop mediated at least in part by increased phosphorylation of AKT and one of its downstream targets, FoxO1, which regulates transcription of *Sox2* [142]. When FoxO1 is phosphorylated, it translocates out of the nucleus, reducing *Sox2* transcription. Thus in ESC, Sox2 can regulate its own expression at the transcriptional levels by both positive and negative feedback loops when Sox2 expression is too low and when Sox2 expression is too high, respectively. Importantly, overexpression of SOX2 in brain tumor cells and PDAC cells from an inducible promoter does not reduce expression of the endogenous SOX2 [104, 114], which suggests that the negative feedback loop is not active in at least some tumor cells. From the discussion in this section, it is evident that the transcriptional regulation of *SOX2* has been extensively studied. However, there is far more to learn regarding how this gene is regulated at the transcriptional level.

### MicroRNAs and SOX2 expression

A large body of data has implicated microRNAs (miRs) in the function of normal embryonic and adult cells, as well as diseased tissues, in particular cancer. More than 10 years ago, ChIP-Chip studies conducted by Boyer et al determined that SOX2 associates with the regulatory regions of many miR genes in human ESC [143]. This finding was extended by ChIP-seq analysis of Sox2 chromatin binding in mouse ESC [144]. More recently, Fang et al determined by ChIP-seq that SOX2 is bound to over 100 miR genes in a glioblastoma cell line [116]. Further study is expected to show that SOX2 regulates the transcription of a large number of miRs in a wide variety of SOX2-positive tumors. However, the specific miR genes regulated by SOX2 are expected to differ widely between tumor cell types due to differences in their transcriptional circuitries.

**Figure 1:**
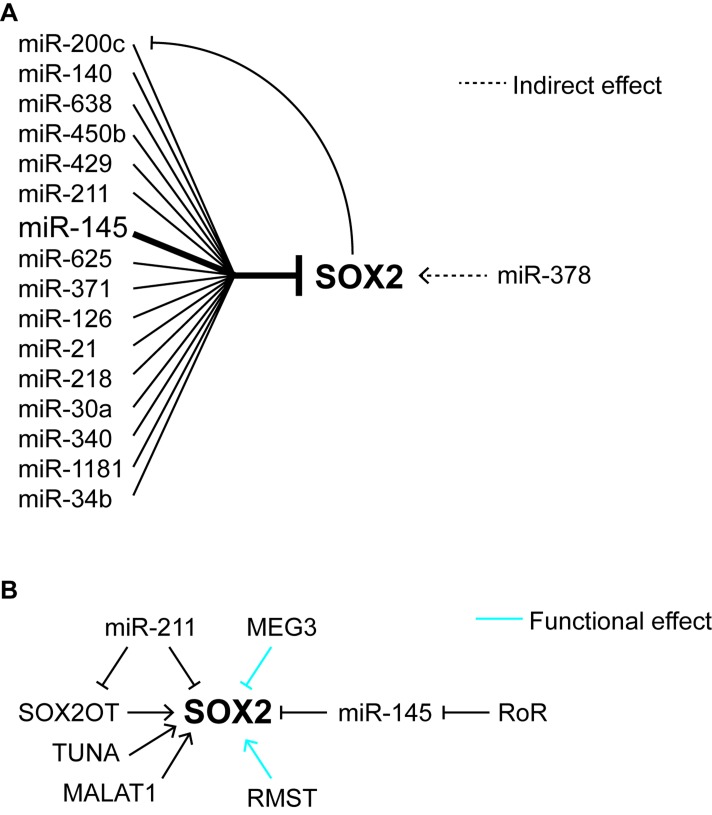
Regulation of SOX2 by miRNAs and lncRNAs **A.** Effects of miRNAs on the expression of SOX2. **B.** Effects of long non-coding RNAs on SOX2 expression and function.

In addition to the regulation of miRs by SOX2, there is a growing list of miRs that are capable of regulating *SOX2* at the post-transcriptional level. In the case of cancer, at least 18 miRs have been reported to regulate SOX2 expression in tumor cell lines (Figure 1A, Table 3). Of these, miR-145 has been implicated directly or indirectly in ESC and at least seven cancers, glioblastoma, prostate cancer, non-small cell lung carcinoma, Ewing sarcoma, hepatocellular carcinoma, PDAC, and urothelial carcinoma [145–152]. Interestingly, in glioblastoma, SOX2 and miR145 have been reported to form a negative feedback loop with one another (Figure 1A). In this tumor, SOX2 can associate with the gene regulatory regions of *miR145*, where it is believed to repress *miR145* transcription; whereas miR145 reduces the expression of SOX2 by interfering with its translation [116]. In colorectal cancer, miR-200c and SOX2 also appear to regulate one another by a negative feedback loop [153].

**Table 3:** MicroRNAs regulating SOX2 in cancer

Tumor Type	miR	Effects Observed	Reference
Breast cancer	miR-140	can target SOX2 3'UTR	Zhang et al., 2012
		altered expression alters SOX2 expression	
	miR-378	Enhances SOX2 expression indirctly	Deng et al., 2013
Colorectal cancer	miR-200c	can target SOX2 3'UTR	Lu et al., 2014
		altered expression alters SOX2 expression	
	miR-638	can target SOX2 3'UTR	Ma et al., 2014
		altered expression alters SOX2 expression	
	miR-450b	targets SOX2 directly	Jin et al., 2016
	miR-429	can target SOX2 3'UTR	Li et al., 2013
		altered expression alters SOX2 expression	
Embryonal carcinoma	miR-211	targets both SOX2 and SOX2OT	Shafiee et al., 2016
Embryonic stem cells	miR-145	targets SOX2 3'UTR upon differentiation	Xu et al., 2009
Esophageal cancer	miR-625	can target SOX2 3'UTR	Wang et al., 2014
		altered expression alters SOX2 expression	
Ewing sarcoma	miR-145	altered expression alters SOX2 expression	Riggi et al., 2010
Gastric cancer	miR-371	can target SOX2 3'UTR	Li et al., 2016
		altered expression alters SOX2 expression	
	miR-126	can target SOX2 3'UTR	Otsubo et al., 2011
		altered expression alters SOX2 expression	
Glioblastoma	miR-21	low miR-21/high SOX2 in one subgroup	Sathyan et al., 2015
		high miR-21/low SOX2 in different subgroup	
	miR-145	SOX2 and miR-145 regulate each other	Fang et al., 2011
Glioma stem cells	miR-218	elevated miR-218-5p reduced SOX2	Wu et al., 2016
		miR-218-5p may not target SOX2 directly	
	miR-9*	ID4 decreases miR-9* and increases SOX2	Jeon et al., 2011
		SOX2 3'UTR activity elvated as ID4 increases	
Hepatocellular carcinoma	miR-126	can target SOX2 3'UTR	Zhao et al., 2015
	miR-145		Jia et al., 2012
Nasopharyngeal carcinoma	miR-30a	targets SOX2 3'UTR	Qin et al., 2015
Neuroblastoma	miR-340	can target SOX2 3'UTR	Daa et al., 2013
		miR-340 gene is methylated in this tumor	
Non-small cell lung carcinoma	miR-638	can target SOX2 3'UTR	Xia et al., 2014
	miR-145	altered expression alters SOX2 expression	Campayo et al., 2013
Osteosarcoma	miR-126	can target SOX2 3'UTR	Yang et al., 2013
		altered expression alters SOX2 expression	
Pancreatic cancer	miR-145	can target SOX2	Sureban et al., 2013
	miR-1181	directly targets SOX2	Jiang et al., 2015
Prostate cancer	miR-145	altered expression alters SOX2 expression	Ozen et al., 2015
	miR-34b	unclear if it directly targets SOX2	Forno et al., 2015
Urothelial carcinoma	miR-145	altered expression alters SOX2 expression	Fujii et al., 2015

With one notable exception, SOX2-targeting miRs are associated with downregulation of SOX2. However, Deng et al reported that miR-378 increases SOX2 expression in breast cancer [154]. Although the types of breast cancer specimens examined were not described, they noted miR-378 was expressed at higher levels in breast tumor tissue than adjacent non-tumorigenic tissue. Rather than directly affecting SOX2, miR-378 targets vimentin, which influences SOX2 expression. The influence of vimentin was shown by overexpression of vimentin and the resulting downregulation of SOX2, but the mechanism by which vimentin regulates SOX2 was not determined. The association of SOX2 and miRs in specific cancers has been inferred predominately from the correlation between elevated SOX2 expression and low miR expression. In most studies, this association is supported by two additional lines of evidence, down regulation of SOX2 when the miR in question is ectopically elevated in tumor cell lines, and down regulation of a reporter gene construct, typically luciferase, containing a portion of the *SOX2* 3’UTR when the miR is ectopically expressed in tumor cell lines (Table 3).

For some cancers only a single miR has been implicated thus far in the regulation of SOX2. For example, miR-30a, when upregulated in nasopharyngeal carcinoma cells, appears to be capable of reducing SOX2 protein by targeting the 3’ UTR of *SOX2* mRNA [155]. However, it is likely that SOX2 can be regulated by several miRs in the same cell type. In gastric carcinoma (see below), prostate cancer, and colorectal cancer, more than one miR has been implicated in the regulation of SOX2 (Table 3). In prostate cancer, SOX2 expression is associated with low expression of both miR-145 and miR-34b [147, 156]. However, unlike miR-145, which has been shown to target the 3’ UTR of the SOX2 transcript [145], it is unclear whether miR-34b targets SOX2 directly. As noted earlier, SOX2 is associated with a higher Gleason score in a subset of prostate tumors that express SOX2 [41]. In the case of colorectal cancer, miR-200c, miR-638, miR-450-5p, and miR-429 have been reported to regulate SOX2, but with different outcomes (Table 3). Lu et al reported that miR-200c, which is expressed at lower levels in colorectal specimens and highly metastatic colorectal cell lines, exhibits an inverse relationship with SOX2 [153]. Similarly, Ma et al has reported that miR-638, which is expressed at a lower level in colorectal tumors than adjacent non-tumorigenic tissue, is able to target SOX2 [157], and Jin et al reported that miR-450-5p, which is downregulated in recurrent colorectal cancer, is capable of downregulating SOX2 [158]. In contrast, Li et al reported that higher levels of miR-429 and lower levels of *SOX2* mRNA in colorectal cancer are correlated with poor survival after surgery [159]. Interestingly, these investigators argued that high miR-429 expression exerts its anti-apoptotic function by downregulating SOX2. However, this is inconsistent with the apparent oncogenic role of SOX2 in a subgroup of colorectal cancer patients. In this regard, Lundberg et al reported that SOX2-positive colorectal cancer patients do not survive as long as SOX2-negative colorectal cancer patients, and this differential is larger for patients with BRAFV^600E^ mutations who survive for substantially shorter periods than those who are SOX2-positive, but lack the BRAF mutation [160]. Going forward, it will be important to determine whether the levels of miR-429 are lower in colorectal cancer patients with BRAFV^600E^ mutations. In view of our earlier proposal that SOX2 levels must be carefully titrated to maximize tumor growth, one of the mechanisms by which miR-429 promotes colorectal cancer may be to help maintain SOX2 within optimal levels for the BRAFV^600E^ mutant subgroup of colorectal tumors.

More than one miR has also been reported to target SOX2 in gastric carcinoma. For miR-371-5p and miR-126, high miR expression is associated with low SOX2 expression. Li et al reported that miR-371-5p, which is elevated in gastric carcinoma compared to adjacent normal tissue, targets SOX2 [161]. In addition, these investigators reported that miR-371-5p downregulated a luciferase reporter gene construct containing a short sequence from the SOX2 3’UTR; whereas blocking expression of this miR in gastric tumor cell line increased SOX2 expression and cell proliferation *in vitro*. A similar conclusion was reached for miR-126. Otsubo et al reported that transiently elevating miR-126 in gastric cancer cell lines decreased SOX2 and increased cell proliferation *in vitro* [162]. They also demonstrated that miR-126 reduced the expression of a luciferase reporter gene containing regions taken from the SOX2 3’ UTR. Furthermore, these investigators reported low SOX2 expression and elevated miR-126 in some gastric tumor specimens, but the results reported do not show a clear pattern. Although, elevated miR-126 expression and low SOX2 expression was observed in several gastric cancer tumor specimens, low miR-126 expression was accompanied by low SOX2 expression in several other gastric tumor specimens. Thus, a larger number of tumor specimens will need to be evaluated to resolve the relationship between miR-126 and SOX2. In addition, the relationship between miR-126, SOX2, and patient survival remains to be determined. As noted earlier, high SOX2 in gastric cancer has been reported to be associated with longer patient survival [31, 42, 56]. Interestingly, there are reports that miR-126 can act as a tumor suppressor in other types of cancer. For example, Yang et al and Zhao et al reported that miR-126 behaves as a tumor suppressor in osteosarcoma and hepatocellular carcinoma, respectively, by targeting SOX2 [163, 164]. Additionally, Hamada et al reported that loss of miR-126 expression is observed in invasive PDAC [165].

Although miRs are recognized as important regulators of SOX2 expression, two important issues need to be considered. First, unless the cell of origin and its expression of miRs have been determined, it remains to be determined whether the miR in question has, in fact, been lost during tumor progression. Second, the full spectrum of SOX2-targeting miRs is likely to be far greater than those already identified.

### Long non-coding RNAs and SOX2 expression

In addition to miRs, several long non-coding RNAs (lncRNAs) have been reported to influence the levels of SOX2 in tumor cells (Figure 1B). LncRNAs are a class of RNAs greater than 200 nucleotides that lack protein-coding sequences. They are transcribed by RNA polymerase II and they are spliced, 5’ capped, and 3’ polyadenylated. The human genome contains several thousand lncRNAs, and there is growing evidence that many play major roles in gene regulation by influencing chromatin structure, gene transcription, and processing of mRNA [166]. More recently, several lncRNAs have been implicated in the regulation of SOX2 expression and its transcriptional activity. The first direct link between SOX2 and lncRNAs was the discovery that the single exon *SOX2* gene is embedded within an intron of a multi-exon lncRNA gene known as *SOX2 overlapping transcript* (*SOX2OT*, also known as non-protein-coding RNA 43) [167]. Like *SOX2* itself, *SOX2OT* orthologues are expressed widely in other vertebrates, including in mouse, chicken, and zebrafish. *SOX2* and *SOX2OT* are both transcribed in the same direction. *SOX2OT* is reported to possess at least 10 exons with up to four different transcription start sites. Through use of alternative transcription start sites and alternative splicing at least 8 splice variants of *SOX2OT* can be generated [168, 169].

*SOX2* and *SOX2OT* have been shown to be co-expressed in ESC, as well as breast, lung, brain, and esophageal tumors [170–174]. In each of these cancers, more than one splice variant is expressed, and the splice variants expressed differ between different cancers. *SOX2* and *SOX2OT* are also likely to be co-expressed in hepatocellular carcinoma. Separate studies have reported that expression of *SOX2* and *SOX2OT* in hepatocellular carcinoma is each associated with poor prognosis [175, 176]. Although the mechanistic relationship between *SOX2* expression and *SOX2OT* remains to be determined, several studies support the conclusion that *SOX2OT* lncRNA contributes to the expression of *SOX2*. Knockdown of *SOX2OT* by siRNA in the lung adenocarcinoma cell line A549 reduced the expression of *SOX2* transcripts [173]. Conversely, forced overexpression of *SOX2OT* in the breast tumor cell line MDA-MB-231 increased the expression of SOX2 transcripts and protein [171]. Intriguingly, *SOX2* and *SOX2OT* expression may both be related by at least one miR. miR-211 has been reported to target the same sequence in transcripts of *SOX2OT* and *SOX2* and lead to their downregulation when miR-211 is overexpressed in the human embryonal carcinoma cell line NT-2 [177]. Thus far, only miR-211 has been reported to downregulate *SOX2* and *SOX2OT*. Future studies should examine whether other miRs that have been shown to target *SOX2* (Table 3) also target one or more of the *SOX2OT* splice variants.

In addition to *SOX2OT*, several other lncRNAs have been directly implicated in the expression of *SOX2*. The lncRNA *TUNA* (Tcl1 Upstream Neuron-Associated), which can form a complex with three RNA-binding proteins, has been shown by performing chromatin isolation *via* RNA purification (ChIRP) to associate with the *Sox2* promoter in mouse ESC [178]. Furthermore, knockdown of *TUNA* by shRNA reduced the expression of Sox2 and led to the differentiation of mouse ESC. Interestingly, *TUNA* and Sox2 are also co-expressed in the brain [178]. Thus, it will be interesting to determine whether *TUNA* is expressed in glioblastoma and medulloblastoma and other SOX2-positive tumors, where it may also contribute to SOX2 expression. However, further study will be needed to determine how *TUNA* influences SOX2 expression.

The lncRNA *MALAT1* (Metastasis-Associated Lung Adenocarcinoma Transcript 1) also appears to influence the expression of SOX2. *MALAT1* has been shown to be expressed in the glioma tumor cell line SHG139S and in two pancreatic tumor cell lines, AsPC1 and CFPAC-1 [179, 180]. Knockdown of *MALAT1* in each of these tumor cell lines reduced the expression of SOX2. However, it is unclear whether the effect of *MALAT1* on SOX2 in these tumor cells is direct or indirect. Equally interesting is the report that lncRNA *RoR* supports SOX2 expression by functioning as a miRNA sponge. Specifically, *RoR* helps maintain SOX2 expression by serving as an RNA decoy that competes for miRs (e.g. miR145) that target *SOX2* expression [181].

LncRNAs also appear to regulate the transcriptional activity of SOX2. The lncRNA *RMST* (RhabdoMyoSarcoma 2-associated Transcript) has been reported to coregulate SOX2 target genes during neurogenesis [182]. *RMST* interacts physically with SOX2 and it promotes the binding of SOX2 to the regulatory regions of neurogenic transcription factors. Impressively, knockdown of *RMST* reduces SOX2 association with approximately half of its chromatin binding sites [182]. Although *RMST* appears to enhance the transcriptional activity of SOX2, at least one lncRNA, *MEG3* (Maternally Expressed Gene 3) that physically associates with SOX2, can interfere with its action. Knockdown of *MEG3* has been reported to increase the association of SOX2 with the *BMP4* gene, which is inhibited by SOX2, and decrease the transcription of this gene [183]. Thus far, the domains of SOX2 that associate with these lncRNA have not been determined, nor has it been determined how they influence the transcriptional activity of SOX2. Going forward, it will be interesting to determine whether *RMST* and *MEG3* are commonly expressed in SOX2-positive tumors, including glioblastoma and medulloblastoma. Moreover, *MEG3* has been shown to be expressed in PDAC cell lines, where its knockdown led to a reduction in cell number *in vitro* [184]. Thus, it will be interesting to determine whether knockdown of *MEG3* alters the function of SOX2 in PDAC cells.

### Post-translational modifications of SOX2

**Figure 2:**
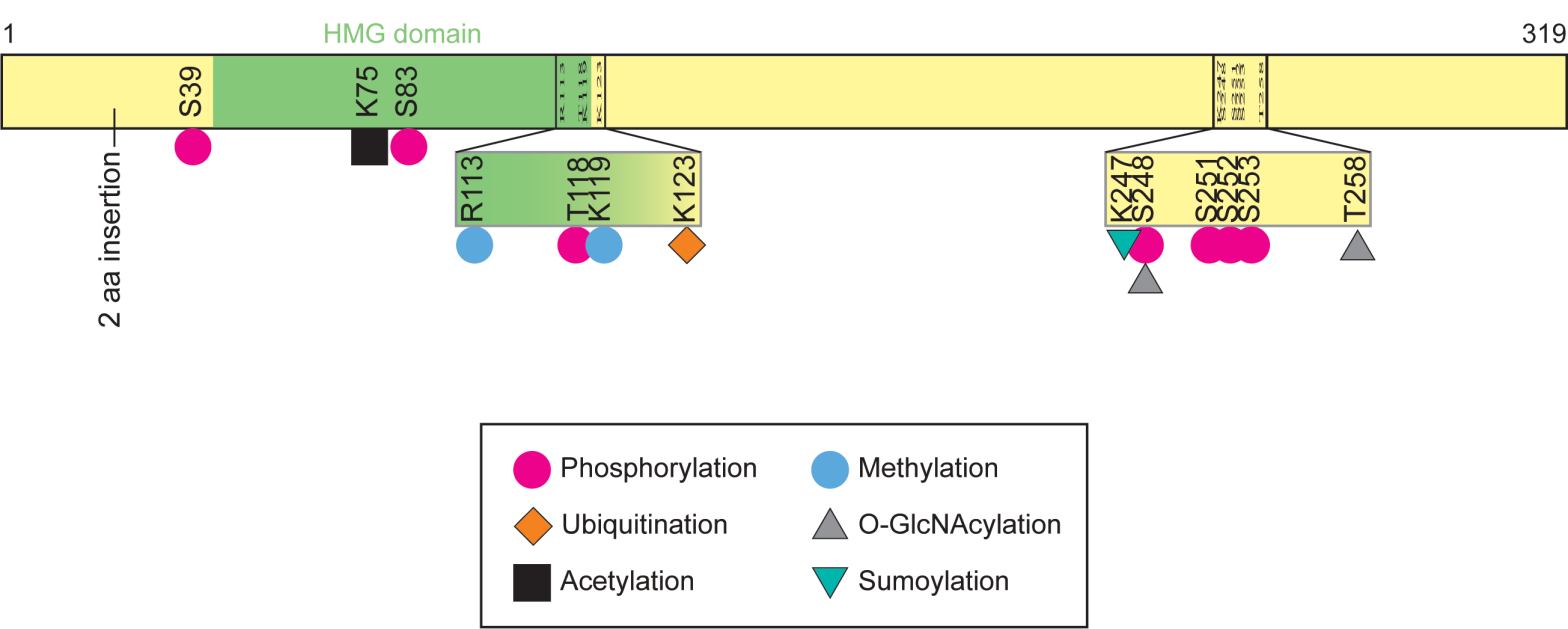
Mouse Sox2 structure and post-translational modification sites Illustration of the 319 amino acid (aa) *Mus musculus* Sox2 and established post-translational modifications. Positions of comparable post-translational modifications in human SOX2 would differ due to the 2 aa inserted at residue 23 in mouse Sox2. Human SOX2 totals 317 aa; whereas mouse Sox2 totals 319 aa.

Another important mechanism used to regulate SOX2, including its transcriptional activity, nuclear localization, and stability, is post-translational modifications, which include phosphorylation, glycosylation, sumoylation, methylation, ubiquitination, and acetylation. Thus far, nearly all reports of SOX2 post-translational modifications have been conducted with mouse pluripotent stem cells and mostly with ESC. In the future, it will be important to characterize the post-translational modifications of SOX2 in tumor cells. Recently, one study described a SOX2 post-translational modification (phosphorylation) in human lung squamous cell carcinoma cells [185]. For the purposes of clarity, and to avoid confusion, the reader is reminded that human SOX2 and mouse Sox2 differ in length by 2 amino acids: 317 amino acids and 319 amino acids, respectively, due to a two amino acid insertion beginning at residue 23 in mouse Sox2 (Figure 2, Table 4).

**Table 4:** Post-translations modifications of mouse Sox2 The sequence numbering shown refers to mouse Sox2 except for human SOX2 T118* (where indicated).

Site	Modification(s)	Effects Observed	Modifying enzyme	Reference
S39	Phosphorylation	Reduces reprogramming	Cdk2	Ouyang et al., 2015
K75	Acetylation	Low level acetylation increases reprogramming. Nuclear export?	likely p300/CBP	Baltus et al., 2009
			Sirt1 (deacetylate)	
S83	Phosphorylation	ND	ND	Malak et al., 2015
R113	Methylation	Increased transcriptional activity, increased self-association	CARM1	Zhao et al., 2011
T118	Phosphorylation	Increased transcriptional activity, increased stability	AKT	Jeong et al., 2010
		Blocks monomethylation at K119	ND	Fang et al., 2014
K119	Methylation	Increases Sox2 ubiquitination	set7	Fang et al., 2014
K123	Ubiquitination	Targets Sox2 for proteasomal degradation	Ube2s	Wang et al., 2016
K247	Sumoylation	Block Sox2's transcription of Fgf2 and Nanog	ND	Tsuruzoe et al., 2006
S248	Phosphorylation	Alter transcriptional activity	ND	Ouyang et al., 2015
	O-GlcNAcylation	Alter transcriptional activity	ND	Myers et al., 2011
				Jang et al., 2012
S251	Phosphorylation	Regulate sumoylation	ND	Tsuruzoe et al., 2006
S252	Phosphorylation	Regulate sumoylation, reduces reprogramming	ND	Tsuruzoe et al., 2006
S253	Phosphorylation	Regulate sumoylation	Cdk2	Tsuruzoe et al., 2006
T258	O-GlcNAcylation	ND	ND	Myers et al., 2011
				Jang et al., 2012
T118* (H)	Phosphorylation	Increase SOX2 transcriptional activity	PKCι	Justilien et al., 2014

Not determined (ND).

The most common and diverse post-translational modification reported for Sox2 is phosphorylation. Sox2 phosphorylation influences its transcriptional activity and its stability. Studies by several research teams have shown that Sox2 can be phosphorylated *in vivo* on at least 6 serine residues (mouse S39, S83, S248, S251, S252, S253) and two threonine resides (mouse Sox2 T118 and human SOX2 T118) (Figure 2) [186–188]. Sox2 has also been reported to be phosphorylated on tyrosine residues when ectopically expressed in 293T cells, which express little if any endogenous SOX2 [186]. It remains to be determined whether these tyrosine residues are phosphorylated in cells that endogenously express SOX2. The kinases responsible for serine phosphorylation of SOX2 have only begun to be determined. For example, Cdk2 can phosphorylate both S39 and S253 *in vitro* [186]. Modifying both serine residues by conversion to alanine (S39A, S253A) reduces the ability of mutant Sox2 to reprogram somatic cells into iPS cells. Surprisingly, even though S39 and S253 are phosphorylated in mouse ESC, and most highly phosphorylated during mitosis, a mutant form of Sox2 (S39A/S253A) is able to support the self-renewal of mouse ESC when endogenous Sox2 is depleted [186]. It is possible that at different levels of Sox2, phosphorylation of these serine residues is dispensable.

Serine 248 of mouse Sox2 has been reported to be phosphorylated in mouse ESC. Phosphorylation of this serine is likely to have a significant role in the function of SOX2, because this serine, along with T258 in mouse Sox2 (see below) can also be modified by *O*-GlcNAcylation [189, 190]. Studies conducted thus far suggest that modification of S248 in mouse Sox2 may alter the transcriptional activity of Sox2, but further work will be needed to properly dissect the impact of phosphorylation and *O*-GlcNAcylation of Sox2 S248. Similar to this serine residue, the serine triplet S249-S250-S251 in human SOX2 appears to regulate another post-translational modification, sumoylation. Human SOX2 has been shown to be sumoylated on K245 and K247 in mouse Sox2 [188, 191]. Importantly, sumoylation of K245 is abolished in the SOX2 mutant (S249A-S250A-S251A) [191]. Thus, phosphorylation of one or more serine residues in the triplet appears to serve as a priming step in the subsequent sumoylation of SOX2. Although the roles of SOX2 sumoylation remain to be fully characterized, sumoylation of mouse Sox2 has been reported to reduce the ability of Sox2 to increase the transcription of *Fgf4* and *Nanog* [188]. In the future, it will be important to determine whether this reduction in transcriptional activity is restricted to a small number of genes or is true for most Sox2-regulated genes.

The kinases that phosphorylate threonine residues of mouse Sox2 T118 and humanT118 have been identified. These threonine residues are both located within a consensus nuclear localization sequence and the HMG domain of SOX2, which is responsible for DNA binding. Phosphorylation of human SOX2 on T118 is mediated by PKCβ [185]. Phosphorylation of this threonine is associated with an increase in the transcriptional activity of SOX2, which was shown using SOX2 mutants. The transcriptional activity observed with wild-type SOX2 was not observed with the SOX2 mutant (T118A), but exhibited by the SOX2 phospho-mimic mutant (SOX2-T118D). Interestingly, human SOX2-T118A does not appear to alter SOX2 stability. In stark contrast, the mouse mutant Sox2-T118A exhibits reduced stability. Mouse Sox2 can be phosphorylated on T118 by AKT in mouse ESC [192]. Phosphorylation of this serine not only increases SOX2 stability, it also increases its transcriptional activity. Remarkably, phosphorylation of mouse T118 blocks the monomethylation of Sox2 on the adjacent K119 by the methyltransferase set7 [193]. Methylation of K119 induces the ubiquitination of Sox2 by the E3 ligase WWP2 and the degradation of Sox2 [193]. Thus, the antagonistic phosphorylation-methylation switch mediated by T118-K119 alters the transcription activity and stability of Sox2, respectively. Importantly, we are not aware of any studies reporting that AKT inhibitors reduce the stability of SOX2 in tumor cells. This warrants attention given the use of AKT inhibitors in many cancer clinical trials. As discussed earlier, AKT has been implicated in a negative feedback loop that influences the transcription of the Sox2 gene in ESC [142].

Sox2 can also be ubiquitinated on lysine K123, which is located just beyond the C-terminal portion of the DNA binding domain of Sox2 (the HMG domain). The ubiquitin-conjugating enzyme E2S (Ube2s) mediates K11-linked polyubiquitination of Sox2 at this site [194]. When ubiquitinated on K123, Sox2 is targeted for proteasome-mediated degradation. The comparable lysine of human SOX2 is K121. Although SOX2-T118A does not appear to be less stable than wild-type SOX2 in lung squamous cell carcinoma cells, it is possible in some tumor cells that phosphorylation of T118 (human SOX2) may block ubiquitination of SOX2 at K121, as was discussed above for T118 (mouse Sox2), and its influence on the methylation of K119 and the subsequent degradation of Sox2.

In addition to Sox2 methylation and *O*-GlcNAcylation discussed above, SOX2 can also be methylated and *O*-GlcNAcylated on other amino acids. Sox2 T258 has been shown to be modified by *O*-GlcNAcylation in mouse ESC. Thus far, the function of T258 *O*-GlcNAcylation has only been studied in the context of double and triple mutants (T258A/S259A and S248A/T258A/S259A). The double mutant reduced the ability of Sox2 to reprogram somatic cells to iPS cells; whereas the triple mutant did not [190]. Additionally, Sox2 can be methylated on R113 by the arginine methyltransferase CARM1, which increases SOX2 self-association and increases the transcriptional activity of Sox2 [195]. However, further study will be needed to determine whether the increase in Sox2 transcriptional activity is linked to its self-association. Furthermore, it is possible that methylation of Sox2 R113 increases its association with other Sox family members [195, 196]. R113, which is located within the HMG domain of Sox2, is located within a second Sox2 nuclear localization sequences (NLS2). However, the Sox2-R113K mutant, which cannot be methylated, did not alter the subcellular location or the stability of Sox2 [195].

Finally, Sox2 has been shown to be acetylated within its DNA binding domain on K75 *in vitro* [197]. Although the acetyltransferase that acetylates Sox2 *in vivo* has not been determined definitively, p300/CBP is a likely candidate, especially since Sox2 can be acetylated by p300/CBP on K75 *in vitro* [197]. Moreover, Sox2 has been shown to recruit p300 to the Fgf4 enhancer in ESC [198]. Blocking acetylation of Sox2 in ESC, as shown with the Sox2-K75A mutant, led to retention of Sox2 in the nucleus and maintenance of its transcriptional activity; whereas, the acetyl-mimic Sox2-K75Q mutant, associates with the nuclear export machinery, specifically Crm1 [197]. Other studies indicate that Sox2 can be deacetylated by Sirt1, a member of the sirtuin family of NAD-dependent protein deacetylases [199, 200]. Acetylation of Sox2 not only affects its function in ESC, a low level of Sox2 acetylation enhances reprogramming of somatic cells to iPS cells [200].

It is clear from the discussion in this section that post-translational modifications of SOX2 dramatically alter its function, and undoubtedly play key roles in helping to adjust the function and levels of SOX2 needed to support cellular activity. However, many questions remain to be addressed. In addition to the enzymes responsible for creating the variety of SOX2 post-translation modifications, enzymes that remove some of these modifications of SOX2 have not been identified. Besides phosphatases, likely candidates include deubiquitinating enzymes (DUBs). Interestingly, proteomic analysis of the SOX2-interactome indicates that SOX2 associates with several DUBs that exert important roles in tumor cells, including USP9X, USP7, USP15, USP24, and USP34 [125, 128, 201]. In the future, defining the roles of each of the SOX2 modifications and the enzymes involved in tumor cells may provide valuable insights into possible strategies for targeting SOX2 in a large number of cancers (Tables 1-2). An equally important question that warrants careful attention is the extent to which any given SOX2 molecule is simultaneously modified by more than one post-translational modification. By analogy to the histone code, a “SOX2 code” of post-translational modifications is likely to play a key role in orchestrating the formation of the multitude of SOX2-protein complexes (SOX2-interactome) needed to properly control the level, transcriptional activity, subcellular localization, and stability of SOX2.

## CONCLUSIONS AND FUTURE PERSPECTIVES

It is evident from work conducted over the past 15 years that SOX2 is far from monolithic. Life is not possible without expression of SOX2, in particular during embryogenesis, but SOX2 also has a dark side. SOX2 is expressed in at least 25 different cancers and in many of these cancers SOX2 expression has been directly implicated in increased tumor growth, metastasis, drug resistance, and poor survival. Thus, targeting SOX2 expression or its mode of action could improve the survival of patients with some of the most difficult to treat cancers.

For the many cancers where SOX2 expression poses a serious threat, much more work needs to be conducted to understand mechanistically how SOX2 contributes to the biology of the tumor. This is particularly evident for the transcriptional regulation of *SOX2*, which has been woefully understudied in cancer biology. Studies conducted primarily in mouse ESC have identified several essential distal enhancers that control *SOX2* transcription. However, the roles of these enhancers in *SOX2* expression in human tumor cells are poorly understood. In addition, ChIP-seq has only been used in two tumor studies to identify genome-wide binding of SOX2 [116, 202]. A similar situation exists regarding the roles of SOX2 post-translational modifications, which, thus far, have only been examined in one human tumor study. Interestingly, studies conducted in ESC indicate that there is significant cross-talk between different post-translation modifications, but this too has not been explored in human tumor cells.

In contrast to our lack of understanding of *SOX2* transcriptional regulation and post-translational modification in human cancer, a significant amount of work has focused on miRs and SOX2 expression in many cancers. Similarly, several lncRNAs have been shown to influence the expression and function of SOX2. Particularly interesting is the ability of SOX2OT to regulate SOX2 expression. However, far more work needs to be performed before the impact of lncRNAs on SOX2 expression is understood in human cancer. Similarly, efforts are needed to determine whether SOX2 regulates the expression of lncRNAs. Given the large number of genes bound by SOX2, it would not be surprising to find that SOX2 influences the expression of many lncRNA, including some that regulate SOX2 expression and function.

Intriguingly, recent studies indicate that small changes in the levels of SOX2 can radically affect tumor cell behavior. Thus far, both small increases and small decreases in SOX2 expression have been shown to adversely influence tumor cell growth in at least five different types of cancer. In the future, it will be important to determine how small decreases as well as small increases in SOX2 expression adversely affect tumor cell proliferation. Finally, we discussed potential problems using cell lines engineered to stably overexpress SOX2. It is evident that the initial response of many, if not most, tumor cells is growth inhibition when SOX2 levels are elevated. Thus, we emphasize the benefit of using cell lines engineered for inducible overexpression of SOX2, both *in vitro* and *in vivo*. Similarly, we have pointed out that markers used in many studies to isolate cancer stem cells may lead to erroneous conclusions and over estimation of the size of the tumor-initiating population.

In conclusion, we believe the work performed thus far indicates that the expression and function of SOX2 in cancer clearly warrants further study. Although significant progress has been made during the past 10 years, far too many questions remain to be answered about SOX2 and this deadly disease. Addressing these questions is expected to lead to new insights into the functions of SOX2 in cancer, which will help design new strategies for more effectively treating cancer.
